# IL-10-Producing ILCs: Molecular Mechanisms and Disease Relevance

**DOI:** 10.3389/fimmu.2021.650200

**Published:** 2021-03-29

**Authors:** Hui Sun, Yuzhang Wu, Yi Zhang, Bing Ni

**Affiliations:** ^1^Department of Pathophysiology, Third Military Medical University, Chongqing, China; ^2^Chongqing International Institute for Immunology, Chongqing, China

**Keywords:** transcriptional regulation, IL-10, ILC2, NK cells, innate lymphoid cells

## Abstract

Innate lymphoid cells (ILCs) are mainly composed of natural killer (NK) cells and helper-like lymphoid cells, which play a vital role in maintaining tissue homeostasis, enhancing adaptive immunity and regulating tissue inflammation. Alteration of the distribution and function of ILCs subgroups are closely related to the pathogenesis of inflammatory diseases and cancers. Interleukin-10 (IL-10) is a highly pleiotropic cytokine, and can be secreted by several cell types, among of which ILCs are recently verified to be a key source of IL-10. So far, the stable production of IL-10 can only be observed in certain NK subsets and ILC2s. Though the regulatory mechanisms for ILCs to produce IL-10 are pivotal for understanding ILCs and potential intervenes of diseases, which however is largely unknown yet. The published studies show that ILCs do not share exactly the same mechanisms for IL-10 production with helper T cells. In this review, the molecular mechanisms regulating IL-10 production in NK cells and ILC2s are discussed in details for the first time, and the role of IL-10-producing ILCs in diseases such as infections, allergies, and cancers are summarized.

## Introduction

The Innate lymphoid cells (ILCs) family consists of natural killer (NK) cells, helper-like lymphoid cells (ILC1s, ILC2s, ILC3s, and lymphoid tissue inducer (LTi) cells) (1). With the exception of NK cells (2), they are mainly flocked on the mucosal surface, which can quickly respond to environmental signals, and then participate in the protection of pathogens, tissue remodeling and tissue homeostasis by producing cytokines and effector proteins (3). However, the dysregulation of ILC function and distribution now appears to be involved in the pathogenesis of a variety of inflammatory diseases, including asthma (4), inflammatory bowel disease (IBD) (5) and psoriasis (6).

IL-10 is an important multifunctional anti-inflammatory cytokine. It targets a variety of innate and adaptive leukocytes, inhibits their activation and function, thus inhibiting the outbreak of inflammatory cytokines, preventing host damage and maintaining the integrity of tissue function. Up to Date, IL-10 is reported to be produced by many different cell types including ILCs, T cells, dendritic cells (DCs), macrophages, mast cells, eosinophils, neutrophils and B cells (7). Among ILCs, NK cells are a major source of IL-10 following infections such as *Toxoplasma gondii*, *Listeria monocytogenes*(Lm), lymphocytic choriomeningitis virus (LCMV) and murine cytomegalovirus (MCMV) (8–11). IL-10-producing NK cells are also found in chronic viral infections and various tumor tissues (12–16). However, in addition to LTi cells, helper-like ILCs have also been reported to have the ability to produce IL-10 under certain conditions, suggesting that IL-10 production is not a unique feature of NK cells. Seehus et al. fist reported that administration of IL-33 induced IL-10 production in mouse lung ILC2s, and they termed this population ILC2_10_s (17), and subsequent studies further indicate that severe and recurrent allergic pulmonary inflammation or asthma are the necessary conditions for the presence of ILC2_10_s (18). However, unlike these pulmonary IL-10-producing ILC2s, which require activation and specific stimulation to produce IL-10, Wang et al. reported that there is a unique subset of IL-10 constitutively expressed in the intestine (19). These intestinal IL-10^+^ ILCs (so-called ILCregs) lack the typical transcription factors of other ILCs and Tregs, such as RORγt, T-bet, Gata3 and Foxp3, but uniquely express Id3 (19).

In brief, lots of evidence suggests that IL-10 expression in ILCs may be very heterogeneous (20). The molecular mechanisms that regulate IL-10 are not fully understood yet, partly because such regulations are cell-type-specific or environment-dependent. In this review, we will summarize the molecular mechanisms regulating the expression of IL-10 in helper-like ILCs and NK cells, and the significance of these IL-10-producing ILCs in immunity and inflammation.

## Molecular Mechanisms Controlling IL-10 Expression in ILCs

### Molecular Mechanisms Controlling IL-10-Producing NK Cells

Natural killer (NK) cells are cytotoxic lymphocytes of the innate immune system, which play a major role in the early control of the virus and microbial infection, tumor growth and metastasis (21). After being activated, NK cells can kill virus‐infected cells or tumor cells by releasing lytic granules containing granzyme and perforin or by inducing death receptor-mediated apoptosis through expression of FasL or TRAIL (22). However, the overactivation of NK cells could lead to severe immunopathological reactions (23–25). To alleviate this danger, NK cells have evolved a subtle mechanism to limit the immune response by producing IL-10. For example, in the context of experimental cerebral malaria (ECM), the immune-dampening effects of NK cells-derived IL-10 are protective to this disease (26).

**Figure 1:**
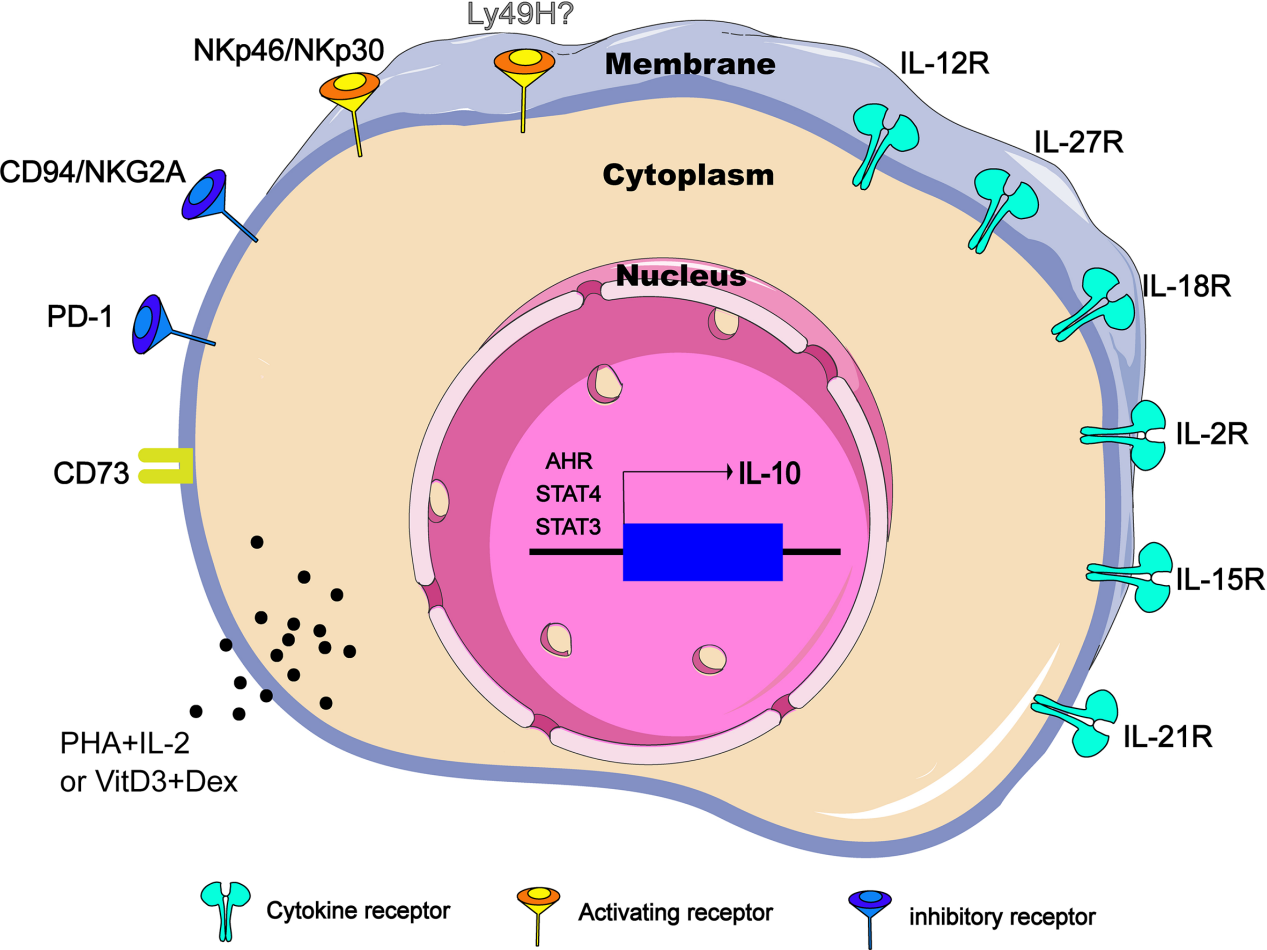
Regulators of IL-10 expression in NK cells. IL-10-producing NK cells can be induced and regulated through different mechanisms, including extracellular chemical factors, cytokine and membrane proteins that trigger signaling, and transcription factors in the nucleus. CD73 is ecto-5’-nucleotidase molecules encoded by *NT5E* gene. PHA, phytohemagglutinin; VitD3, vitamin D3; Dex, dexamethasone.

However, NK cells do not express IL-10 constitutively. In different diseases and conditions, NK cells can regulate the secretion of IL-10 through different ways. It has been clarified that cytokines including IL-12, IL-15, IL-18 and surface receptors such as CD73 and NKG2A are involved in the production of IL-10. Under certain pathological circumstances, several of these pathways play a non-redundant role because any defections of these pathways are enough to change the inhibitory function of NK cells. This section reviews cytokines, cell surface receptors and transcription factors related to IL-10 expression in NK cells in an attempt to reveal the functional plasticity of IL-10-producing NK cells (**Figure 1**).

#### Cytokines That Regulate IL-10-Producing NK Cells

IL-12 cytokine family belongs to type I cytokine super family and consists of IL-12, IL-23, IL-27 and IL-35 of heterodimeric cytokines formed by two subunits, an α-chain (p19, p28 or p35) and a β-chain (p40 or Ebi3) that induce NK effector function both independently and in cooperation with other cytokines (27). IL-12 is the first reported cytokine that induces NK cells to produce IL-10 *in vitro* (28). In the *T. gondii*-infection murine model, IL-12Rβ2^-/-^ NK cells failed to produce IL-10, and NK cells sorted from naive C57BL/6 mice released significant amounts of IL-10 only when stimulated by the addition of IL-12 (8). Therefore, at least in some parasite infections, IL-12 is necessary and sufficient to induce NK cells to produce IL-10 *in vivo* (**Figure 1**). Interestingly, the IL-10 produced by NK cells appears to be related to the intensity of the IL-12 response. When mice are challenged with pathogens that do not lead to disseminated infection, such as influenza virus (intranasal instillation) or an attenuated nondisseminating Yersinia pestis strain (intranasal instillation), NK cells secrete IFN-γ, but no IL-10. However, NK cells will resume production of IL-10 after influenza virus infection supplemented with rIL-12 or after a systemic infection caused by intravenous injection of the pestis strain (8). Also as a member of the IL-12 cytokine family, IL-27 is generally produced by DCs that has received signals through TLRs, IFN-α or IFN-γ (29). In the autoimmune uveitis murine model, NK-DC interaction triggers a positive feedback loop of IFN-γ and IL-27 production. Interestingly, in the co-cultures of LPS–DC and NK cells, not only IFN-γ and IL-27 but also IL-10 was secreted into the supernatant, and IL-10 level was reduced by neutralization of IL-27. Addition of IL-27, but not IFN-γ, to NK or DC cultures was able to stimulate IL-10 production from both cell types, indicating that induction of IL-10 is a direct effect of IL-27 (30) (**Figure 1**).

IL-18 is a member of IL-1 family and was initially identified as an IFN-γ-inducing factor that can co-stimulate Th1 type inflammatory response (31). During severe systemic infections caused by *Listeria monocytogenes*, independent of IL-12 and STAT4, the IL-18 signal empowers NK cells to produce IL-10(**Figure 1**). Importantly, Nlrp3 expression of DCs is required for IL-18 release in response to the *Listeria monocytogenes* p60 virulence protein, because mice lacking *Nlrp3*, *IL-18* or *IL-18R* cannot accumulate IL-10 in the serum (9). It is worth noting that without other DC factors, only IL-18 cannot induce IL-10 production in NK cells, and IL-12 -induced IL-10 secretion does not require IL-18, and blocking IL-12 or STAT4 does not inhibit NK cell IL-10 production induced by DC stimulated by Lm/L1S. Thus, there is an unknown second factor cooperating with IL-18 to promote NK cells to produce IL-10. In conclusion, it is necessary but not sufficient for IL-18 to induce IL-10 production by NK cells in the Lm infection model.

IL-2, IL-7, IL-15 and IL-21 belong to the common cytokine receptor γ chain (γ_c_) family, which plays a central role in both development and functional activities of NK cells (32). Among them, IL-15 has attracted much attention due to its therapeutic potential in cancer (33, 34). Recently, in murine models of Lm infection and experimental cerebral malaria (ILM), it was reported that IL-15 cytokine/receptor complex (IL15C) produces IL-10 by driving STAT3 phosphorylation in NK cells (26, 35) (**Figure 1**). Specifically, STAT3 signal transduction and NK cells IL-10 production are detrimental to host innate resistance to Lm, but may contribute to the survival of cerebral malaria models. It should be noted that, similar to IL-18, the addition of IL-15C (or free IL-15) alone is not sufficient to stimulate the production of IL-10 in cultured NK cells (26). During Lm infection, p-STAT3 was still observed in NK cells of NKIL15Ra^-^ mice (35), suggesting that there are some other factors that co-drive the production of IL-10 by NK cells. IL-18 is one of the possible factors, because IL-18 can effectively assist some cytokines, including IL-15, to drive the activation of STAT3 in NK cells (36, 37), and STAT3 signal is essential for the production of IL-10 in NK cells. IL-21 has a similar molecular structure to IL-15 (38). During murine cytomegalovirus (MCMV) infection, NK cells respond to IL-21 stimulation by producing IL-10 *in vitro*. However, IL-21 is not individually required for infection-induced NK cell IL-10 *in vivo* (39) (**Figure 1**).

In addition, it has been demonstrated that PHA and IL-2 stimulation, vitamin D3/dexamethasone and anti-CD2/CD16 mAb can induce IL-10 expression in NK cells *in vitro* (40) (**Figure 1**), but the exact mechanisms remain to be clarified.

#### Surface Molecules That Regulate IL-10-Producing NK Cells

In addition to cytokine-mediated signal transduction, signal transduction triggered by other cell surface receptors (such as NKG2A) may enhance IL-10 production in NK cells. So far, dozens of NK cell receptors have been discovered. According to their functions, they can be divided into inhibitory receptors and activating receptors. Inhibitory receptors mainly include KIR2DL1 (CD158a), CD94/NKG2A (CD94/CD159a), ILT2/LIR-1 (CD85J) and PD-1 (CD279). The activation receptors mainly include KIR2DS1 (CD158h), KIR2DL4 (CD158d), NCR (NKp46, NKp44 and NKp30), NKG2C (CD159a), 2B4 (CD244) (41). In viral infection or tumor microenvironment, NK cells become exhausted (42–44), and the up-regulated inhibitory receptor is closely related to the production of IL-10 by NK cells. In a report analyzing IL-10 secreting and non-secreting NK cells, it was found that most IL-10 secreting NK cells are limited to the inhibitory KIR-expressing NK cell subsets (40). Specifically, the classic NK cell inhibitory receptor, CD94/NKG2A, has been reported to mediate the production of IL-10 in hepatitis C virus (HCV) infection (45) (**Figure 1**). Recently, it was reported that NK cells express high levels of PD-1 and CD94 in chronic hepatitis B virus (HBV) infection. Correspondingly, HBV-treated monocytes induce NK cells to produce IL-10 through PD-L1 and HLA-E ligand signals (13). However, there appear to be exceptions. For instances, Ly49H is an important activating receptor for mouse NK cells, high IL-10 is being produced in the *Prf1*^−/−^ but not the *Ly49h*^−/−^*Prf1*^−/−^ mice at intermediate times after MCMV infection (46). Conversely, a recent study indicates that Ly49H signals are not necessary because both Ly49H^+^ and Ly49H^-^ NK subsets have the ability to produce IL-10 during MCMV infection. The ability of NK cells to produce IL-10 is mainly caused by the cell proliferation effect mediated by Ly49H (39). In addition, NKp46 and NKp30 may be another special set of activating receptors that can make NK cells produce IL-10 (**Figure 1**). When NK cells are freshly isolated from HCV-infected patients, after stimulation with PHA or anti-CD16, NK cells can be observed to produce IL-10. When stimulated with anti-NCR mAb (NKp46 or NKp30), the level of IL-10 produced will be similar to the level induced by anti-CD16 mAb (14).

CD73 is a metabolic immune checkpoint responsible for coordinating the level of extracellular adenosine, which can control the inflammatory response in the microenvironment of tissues that are stressed or damaged (47). In a variety of tumors, dysregulations of CD73 expression and activity have been reported (48–50). A recent study points out that tumor-infiltrating NK cells up-regulate the expression of CD73, and these CD73^+^ NK cells will undergo transcriptional reprogramming, and increase the production of IL-10 by up-regulating the transcription activity of STAT3, thereby inhibiting the activity of CD4^+^ T cells (15) (**Figure 1**).

#### Transcription Factors That Regulate IL-10 Producing NK Cells

It has been determined that STAT3 activation is closely related to the production of IL-10 (51–53). Whether IL-15C treatment or during systemic Lm infection, NKSTAT3^-^ mice do not produce IL-10 efficiently, and there is ample evidence that serum IL-10 levels in NKSTAT3^-^ mice are similar to those in B6.*IL-10*^-/-^ mice despite Lm infection (35). Similarly, in NK-tumor cocultures, low-dose GPB730 (selective STAT3 inhibitor) pretreatment of NK cells can also inhibit the production of IL-10 (15). Therefore, the activation of STAT3 is necessary for the production of IL-10 by NK cells (**Figure 1**), at least in the immune response to cancer and infection.

The IL-12-STAT4 axis is also involved in the regulation of IL-10 expression (54, 55). In NK cells, IL-2 induction of IL-10 is essentially intact in the absence of STAT4 (**Figure 1**), but IL-12 fails to induce IL-10 expression in *STAT4^−/−^* NK cells. Obviously, STAT4 is the main requirement for IL-10 induced by IL-12 (56). Furthermore, a conserved STAT4 DNA-binding element has been identified in NK cells, which is located in the fourth intron (CNS + 3.10) of the *IL-10* gene in mice and humans. The CNS + 3.10 region containing the STAT4 site is the target of cytokine to induce chromatin remodeling by enhancing the level of histone H3 acetylation (56). However, the production of IL-10 by NK cells appears to be preprogrammed before the IL-12-STAT4 axis is involved in the regulation of *IL-10* genes. Using mitomycin C (MMC) to treat leukocytes from high-dose-MCMV-infected IL-10-GFP-reporter mice at d0, d1.5, d2.5 and d3.5 post infection, respectively, Tarrio ML et al. find the MMC effects for inhibiting IL-10-GFP expression in response to IL-12 are highly significant in cell populations from d0 or d1.5-infected mice but insignificant in populations prepared on d2.5 or d3.5 post infection (39). Therefore, NK cells acquire the IL-10 response to IL-12 only after proliferation and division. Together, regarding to the regulation of IL-10 production by NK cells, the internal regulation of cell proliferation such as the epigenetic modification of histones might be more important than the mediation of IL-12-STAT4 axis.

Other factors such as aromatic hydrocarbon receptor (AHR) signals can also mediate the production of IL-10. AHR is a ligand-activated transcription factor that can interact with various ligands. For example, AHR interacts with the transcription factor c-Maf to promote IL-10 expression in Th1 cells (57). NK cells basically express the Ahr transcript, which can be further induced by IL-12. The IL-10 produced by the *in vitro* expanded NK cells can be elevated by enhancing AHR activity, but would be reduced in the presence of AHR inhibitors. In addition, NK cells isolated from *Ahr*^-/-^ mice infected with *T. gondii* show defects in IL-10 expression. Thus, AHR is identified as a key cofactor involved in IL-10 production by NK cells (58) (**Figure 1**).

### Molecular Mechanisms Controlling IL-10-Producing Helper-Like ILCs

When activated by certain stimuli, all helper-like ILCs, in addition to their signature cytokines, may also secrete IL-10 (59), a condition also seen in highly heterogeneous Tr1 cells (60). However, compared to Tbet and RORγt, GATA3 specifically binds to and promotes the remodeling of the *IL-10* locus (61, 62), so ILC2s appear to produce IL-10 more readily than ILC1 and ILC3s, which partially explains why stable expression of IL-10 is only observed in ILC2s. ILCreg may be an exception, but unfortunately its existence is still controversial. In the following section, we will mainly focus on the regulation of cytokines, adhesion molecules and transcription factors on the production of IL-10 by ILC2s.

#### Cytokines and Stimulus That Regulate IL-10-Producing Helper-Like ILCs

**Figure 2:**
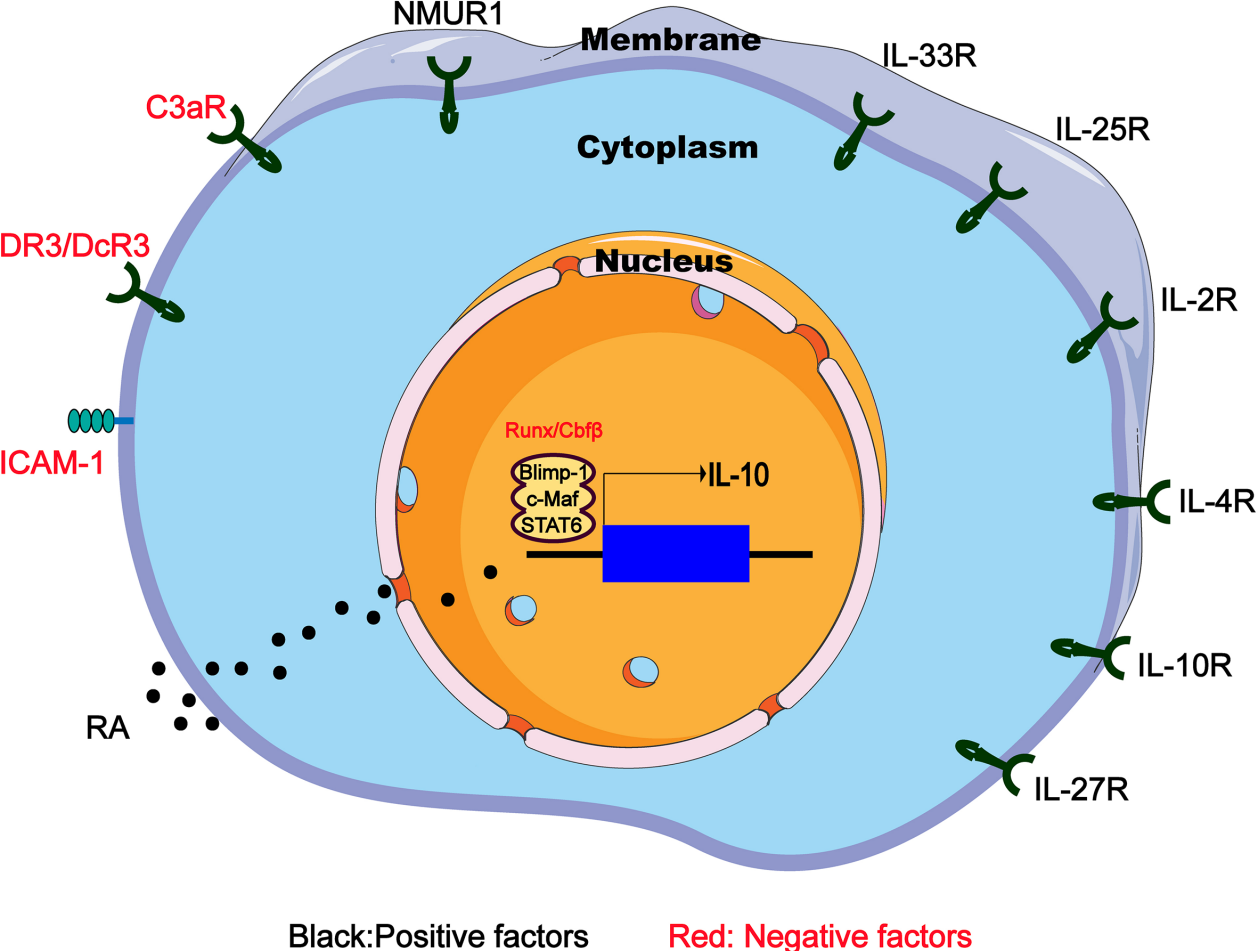
Regulators of IL-10 expression in IL-10-producing ILC2s. IL-10-producing ILC2s can be induced and regulated through different mechanisms including extracellular chemical factors, cytokine and membrane proteins that trigger signaling and transcription factors in the nucleus. RA, retinoic acid; NMUR1, neuromedin U receptor 1; DR3/DcR3, death receptor 3 and decoy receptor 3; ICAM-1, intercellular adhesion molecule-1.

It is known that IL-33 can effectively induce ILC2s to produce IL-10, and such a group of IL-10-producing ILC2 has specific gene expression profile similar to naive ILC2s, which are called ILC2_10_s (17). RNA sequencing of ILC2s in the lung tissue of IL-33-treated mice revealed the increased expression of inhibitory genes such as *IL-10*, *Ctla4* and *Tigit*. Further research showed that after IL-33 injection, the proportion of ILC2_10_s in the CD45^+^ IL-10-producing cells of the hematopoietic compartment increased from 0.7% to 43.9% (17). Other stimulating factors such as IL-2 and retinoic acid (RA) can significantly increase the efficiency of IL-33 to induce IL-10 (**Figure 2**). It is worth mentioning that Morita et al. have demonstrated that RA can induce ILC2s to effectively produce IL-10 in a dose-dependent manner in the presence of IL-2 and IL-33. This group of IL-10–producing ILCs has a phenotype that is more similar to Treg cells rather than ILC2s, so they name these cells ILCregs (63), although this naming is still controversial so far.

IL-4 is reported to play a key immunomodulatory role in many diseases (64, 65). Adoptive transfer of IL-4-induced M2 macrophages secreting IL-10 prevents type 1 diabetes in NOD mice (66). Recently, an interesting experiment demonstrates that IL-4 has the ability to induce the differentiation of ILC2_10_s from the activated ILC2s (18). After IL-10^GFP^ mice were given rmIL-33 intranasally for 3 days to activate lung ILC2s, the IL-10^GFP–^ILC2s were then isolated and cultured for 48 hours with rmIL-2, rmIL-7 and rmIL-4. Surprisingly, an average of 50% of the cultured ILC2s became ILC2_10_s (18). Similarly, culturing intestinal ILC2s *in vitro* also confirm that IL-4 is a positive regulator of IL-10 production (67) (**Figure 2**).

Other factors such as IL-10, IL-27, and neuromedin U (NMU), have also been shown to induce IL-10 production by intestinal ILC2s (68) (**Figure 2**). It has been previously reported that NMU combined with IL-25 expand the inflammatory ILC2s (68). However, NMU can induce ILC2s to produce IL-10, which may be a negative regulation mechanism, indicating the complicated crosstalk between the nerve and the immune system in the allergic inflammation of the mucosal surface. Interestingly, IL-10 can directly act on T cells to inhibit the differentiation and function of Th2 cells (69). IL-10 produced by Th2 cells may play a key self-negative regulatory role in type 2 immune responses (70). However, the situation in IL-10-producing ILC2s is different. IL-10 secreted by ILC2s may activate a positive feedback loop that promotes IL-10 production. After treating cultures containing ILC2s and IL-10 positive modulators (IL-2, IL-4, IL-10, IL-27, and NMU) with IL-10-blocking antibodies, IL-10 expression in ILC2s was significant decrease, indicating that ILC2s themselves can act to amplify IL-10 signaling by secreting IL-10 (67).

Finally, it is worth mentioning that C3a (71) and the TNF superfamily member TL1A are inhibitors of IL-10 production by ILC2s (**Figure 2**). It was previously known that C3a has an inhibitory effect on IL-10 production (70), and significant up-regulation of *IL-10* mRNA expression was observed in C3ar1^−/−^ILC2s (72). Similarly, TL1A promotes allergic immunopathology by activating ILC2s (72), but *in vitro* TL1A strongly eliminates IL-10 production induced by IL-2 or IL-4 (68).

#### Surface Molecules That Regulate IL-10-Producing Helper-Like ILCs

Intercellular adhesion molecule-1 (ICAM-1 or CD54) mainly interacts with leukocyte function-related molecules (LFA)-1 (73) to mediate adhesion reactions (74). In addition to playing a role in mediating the adhesion of inflammatory cells to vascular endothelium, epithelium and extracellular matrix, ICAM-1 also acts as a costimulatory molecule, assisting in the close interaction between cells and signals from the inside out divert (75). For example, costimulation of ICAM-1 by LFA-1 causes T cell activation during antigen presentation (74). It has been clear that ICAM-1 is necessary for the development and function of ILC2s. The ERK signaling pathway in ICAM-1^-/-^ILC2s is impaired, which leads to a decrease in GATA3 protein levels, and the response to allergens reduces the production of type 2 cytokines (75). Further research indicated that the ICAM-1 defect on ILC2s significantly affects its effector functions by down-regulating pro-inflammatory cytokines (such as *Il5*, *Il9*, *Il13*, and *Csf2*) but significantly up-regulating IL-10 level (76). Therefore, ICAM-1 may inhibit the IL-10 production of ILC2s through an unknown regulatory mechanism (**Figure 2**), and further research may find new directions for ILC2-dependent diseases such as allergic asthma.

#### Transcription Factors That Regulate IL-10 Producing Helper-Like ILCs

The transcription factor c-Maf is one of the most important regulators of IL-10 production in Th1, Th2 and Th17 cell subsets, as well as regulatory T cells, macrophages and B cells (77–79). In the case of Toxoplasma *gondii* infection *in vitro* and *in vivo*, Blimp-1 will promote the production of IL-10 in Th1 cells together with IL-12-STAT4 signal, but requires the assistance of cMaf. Interestingly, Blimp-1, STAT4 and c-Maf jointly bind to the DHS-9 region of the *IL-10* locus, although STAT4, not Blimp-1, regulates histone modifications in the DHS-9 region (80). In addition, Blimp-1 regulation has also been associated with Stat1/3 pathways (80, 81). In summary, these studies have determined that the transcription factors cMaf and Blimp-1 are active master regulators of IL-10 production. In ILC2s, cMaf and Blimp-1 are also essential for the production of IL-10, because knocking out any of them leads to a significant decrease in ILC2_10_s frequency (18). Interestingly, this process needs to be mediated by the IL-4-STAT6 axis instead of the IL-12-STAT4 axis, because compared to the control group, IL-10 secreted by ILC2s cells lacking STAT6 is significantly reduced (18). Therefore, IL-4 receptor and downstream STAT6 transcription factor up-regulate cMaf and Blimp-1 transcription factors to produce IL-10 in ILC2_10_s (**Figure 2**).

In chronic or severe airway anaphylaxis, a group of TIGIT^+^IL-10^+^ILC2s has been identified as “exhausted-like” ILC2s because of their high expression of inhibitory receptors such as TIGIT and PD-1, similar to exhausted CD8 T cells (82). Interestingly, the absence of Cbfβ, the binding partner of all Runx proteins in ILC2s, or deficiency Runx1 and Runx3 increases the presence of TIGIT^+^IL-10^+^ILC2s in allergic inflammation *in vivo* and *in vitro (*83), leading to a reduction in allergic inflammation. ILC2s lacking Cbfβ are somewhat similar to ILC2_10_s because the expression of *Tnf*, *Lta*, *Retnla* and *Ccl1* is reduced in both cell types, and IL-10 production is increased (83). However, Cbfβ knockout leads to more overall defects in ILC2 function, because ILC2 lacking Cbfβ cannot well express the main effector cytokines (83), such as *IL-5* and *IL-13*, while ILC2_10_s produces large amounts of IL-5 and IL-13. In addition, the expression of T cell exhaustion markers on ILC2_10_s did not increase, while the high Id3 expression observed in ILC2_10_s was not induced in Cbfβ-deficient ILC2s. Therefore, ILC2s lacking Cbfβ and low response seems to be different from ILC2_10_s (**Figure 2**). However, it is still necessary to further identify and determine the relationship between the both cell types, because it is presently difficult to determine whether they are two independent subsets under different stimuli or just different states of cell fatigue and low responsiveness.

#### Id3 and ILCregs

An important question that remains to be answered is whether Id3^+^ regulatory ILCs (ILCregs) are widespread or not. Earlier, Wang et al. identified a group of regulatory subgroups of ILC (called ILCregs) in the intestines of mice and humans. ILCregs only express transcription factors such as Id3 and Sox4, but lack the typical transcription factors of other ILCs and Treg cells, such as *Rorc* (encoding RORγt), *Tbx21* (encoding T-bet), *Gata3*, and *Foxp3*. In short, ILCregs have a unique gene identity that is characterized by the expression of Id3, which is different from ILCs or Treg cells (19). Subsequently, the same team analyzed the tumor-infiltrating ILCs in the progression of colorectal cancer (CRC) through single-cell RNA sequencing, and also identified the existence of Id3^+^ILCregs with independent functions. In addition, they also found that ILC3s can be transformed into ILCregs during the progression of CRC, which is mediated by TGF-β signaling (84). However, Bando et al. questioned the existence of ILCregs because they were unable to find evidence of the existence of ILCregs in the mouse intestine in various conditions. Instead, they believe that ILC2s are the predominant source of intestinal ILC-derived IL-10, and ILCregs are not a common immune cell population in mice (67).

The existence of Id3^+^ ILCregs is very controversial so far because only one group reports them but other groups have failed to identify them in the intestine or lung of mice. Id3 is part of the inhibitors of differentiation (Id) proteins family, which is a class of negative regulatory nuclear transcription factors (85). As a transcriptional regulator that prevents stem cell differentiation and promotes cell cycle progression, Id3 extensively regulates cell growth, self-renewal, aging, angiogenesis and neurogenesis (86). It has been found that Id3 can positively regulate the differentiation of Treg cells, and the level of Treg in Id3 knockout mice is reduced (87). Further studies have shown that the balance of Id3 and E47 controls the maintenance of Foxp3 expression in Treg cells, thus contributing to the plasticity of Treg cells (88). Interestingly, high expression of Id3 was also found in ILC2_10_s (17). On the other hand, Id3 also regulates the production of IL-10. In the tumor microenvironment, the overexpression of Id3 significantly enhances the production of IL-10 in monocytes, while Id3 knockdown eliminated this effect through specific siRNA (89). In brief, according to the published studies, some subsets of IL-10-producing cells may require Id3. However, it should be noted that Morita et al. could not detect Id3 expression in the IL-10^+^ ILC2s that they term ILCregs (63). Therefore, whether Id3^+^ ILCregs really exist and what role does Id3 play in ILCs still remain to be clarified in the future. Other factors also need to be considered, such as the microbiota, which has been shown to affect the production of cytokines in T cells (90), and the interaction between the microbiota and ILCs is also being widely discussed (91). However, whether the generation of Id3^+^ ILCregs in the intestine requires specific microbiota is worthy to be explored yet. Finally, the influence of the medium on IL-10 production also needs to be considered. It has been reported that IL-10 production of ILC2_10_s depends on the availability of glucose and the completion of the function of the glycolytic pathway (18). However, whether the IL-10 production of Id3^+^ILCregs shares similar metabolic requirements with ILC2_10_s, or requires a unique metabolic pathway needs further verification.

## Il-10-Producing ILCs in Diseases

### IL-10-Producing NK Cells in Diseases

IL-10-producing NK cells play a negative regulatory role during pathogen infection. NK cells are the main source of IL-10 after *Toxoplasma gondii*, Lm, LCMV and MCMV infections (92). The production of IL-10 by NK cells can help reduce pathological immune damage caused by pathogens. For instance, IL-10-producing NK cells induced by IL-15C can prevent the obvious pathology and death in experimental cerebral malaria (92). However, in most cases, IL-10-producing NK cells seem to be harmful. For example, during Lm, *Leishmania* and *Toxoplasma* infections, the secretion of IL-10 may limit the protection of the host by inhibiting the recruitment or activation of inflammatory myeloid cells (35). Similarly, during human virus infection, the appearance of IL-10-producing NK cells may lead to a tilt of the adaptive immune response and the loss of virus control. Compared with HCs, antiretroviral treatment (ART)-naïve HIV-infected patients had increased percentages of IL-10^+^ (2.0% vs. 0.4%, p = 0.015) and TGF-β^+^ (4.5% vs. 2.1%, p = 0.022) NK cells, and ART-treated patients also had a higher percentage of IL-10^+^ NK cells (2.5% vs. 0.4%, p = 0.002) (12). In chronic HBV infection, compared with healthy individuals, NK cells express higher levels of PD-1, CD94 and IL-10 (13), which is adverse to the immune response of T cells to HBV virus.

IL-10 produced by NK cells can inhibit the function of effector T cells and reduce the efficiency of anti-tumor immunity. It has been reported that the level of IL-10 produced by NK cells in a variety of tumors is up-regulated (21). For example, NK cells in pancreatic ductal adenocarcinoma (PDAC) showed reduced cytotoxic activity and low levels of IFN-γ expression, but on the contrary produced high levels of intracellular IL- 10 (93). Similarly, the number of circulating HLA-G^+^IL-10^+^TGF-β^+^NK cells is increased in breast cancer patients (16). In short, IL-10-producing NK cells help cancer cells escape immune.

### IL-10 Producing Helper-Like ILCs in Diseases

A study on murine airway allergy showed that *in vivo* generation of ILC2_10_s could effectively inhibit allergic airway inflammation by reducing the recruitment of eosinophils (17). Recently, an allergen-specific immunotherapy (AIT) cohort control study for patients with house dust mite allergic rhinitis found that the percentages of IL-10^+^CTLA-4^+^ILCs in the responders after 2 years of AIT were increased by 3.2% (95% CI = 0.7%-5.7%) and were higher than in the nonresponders (–0.9% [95% CI = –4.5% to 2.7%])) and placebo-treated patients (1.2% [95% CI = –2.4% to 4.8%]), IL-10–producing innate lymphoid cells increased in patients with house dust mite allergic rhinitis following immunotherapy (94). In addition, in a prospective, double-blind, placebo-controlled trial, Golebski et al. reported that, compared with healthy subjects, patients with grass-pollen allergy had lower frequency of IL-10^+^KLRG1^+^ILC2s, and patients who received grass-pollen sublingual immunotherapy restored the ability of ILC2 to produce IL-10 (95). These clinical studies suggest that IL-10-producing ILC2s may be a potential new target for the treatment of allergic airway inflammation. Besides, recent studies have also shown the protective role of IL-10-producing ILC2s in transplant rejection. Two subsets of ILC2s were identified in islet allografts of IL-33-treated mice: IL-10-producing ILC2s (termed ILC2^10^) and non-IL-10 producing ILC2s (termed non-ILC2^10^). Intravenous transfer of ILC2^10^, but non-ILC^10^, markedly prolonged islet allograft survival in an IL-10-dependent manner (96).

Research on ILCregs is limited. Wang et al. showed that ILCregs inhibit the activation of ILC1s and ILC3s through IL-10 signaling, thereby alleviating intestinal inflammation. Deficiency of Id3 abrogated ILCregs population and displayed severe colitis, which could be attenuated by rescue of WT ILCregs (19). Wang et al. also found that, as colorectal cancer (CRC) tumor progresses, ILCregs in tumor-infiltrating ILCs will gradually increase, and depletion of ILCregs significantly inhibits tumor growth (97). ILCregs can produce a large amount of IL-10 and participate in the immunosuppression of tumor progression.

## Conclusions and Prospection

How to maintain the balance between inflammation and anti-inflammatory lymphocytes is essential for providing effective host defense against pathogen invasion and preventing chronic inflammatory diseases. IL-10 is an important anti-inflammatory cytokine. Although IL-10 can be expressed by many cell types, ILCs are a new source of IL-10 production. Except for LTi cells, all ILCs can express IL-10 under certain stimulation. The IL-10-producing ILCs are very heterogeneous, which could include NK subset, ILC2 subset and ILCregs. This article elaborates on the various molecular mechanisms that are currently known or suggest to regulate the production of IL-10 by NK cells and ILC2s, and their roles in diseases. However, there are still several issues to be addressed urgently: (1) are ILC2_10_s a different subset of ILC2s in different settings and models outside of IL-33 injection? (2) Can other groups confirm the existence of Id3^+^ILCregs? Furthermore, although IL-10 produced by ILC2s may be a stable phenotype, ILC1s and ILC3s cells can also express IL-10 under certain stimulation, which indicates that there is an unknown regulatory IL-10 switch. Cell-type-specific IL-10 regulation may depend on differences in chromatin accessibility in different regulatory regions of the *IL-10* locus in different cell types. Transcription factors may influence this epigenetic difference (60). The epigenetics of the *IL-10* gene has been fully elucidated in Th cells. However, related research in ILCs, especially helper-like ILCs, remains to be elucidated yet. Finally, given that Bhlhe40 is widely involved in IL-10 transcriptional regulation in Th cells (98), however whether it is also involved in the regulation of ILCs remains to be clarified.

## Author Contributions

HS drafted the review manuscript. YZ drew the figures. YW and BN reviewed and finalized the manuscript. All authors contributed to the article and approved the submitted version.

## Conflict of Interest

The authors declare that the research was conducted in the absence of any commercial or financial relationships that could be construed as a potential conflict of interest.
